# EXPLORING THE RELATIONSHIP BETWEEN HOME TIME AND SERIOUS ILLNESS AMONG COMMUNITY-DWELLING OLDER ADULTS

**DOI:** 10.1093/geroni/igae098.2497

**Published:** 2024-12-31

**Authors:** Rachel Butler, Howard Degenholtz

**Affiliations:** University of Pittsburgh, Pittsburgh, Pennsylvania, United States; University of Pittsburgh, Pittsburgh, Pennsylvania, United States

## Abstract

Population-based measures like Healthy Days at Home (HDAH) are increasingly used as quality of life or health system performance metrics. These measures are just beginning to be studied in the context of older adults living with serious illness (SI). We explored factors associated with the duration of home time among community-dwelling Medicare beneficiaries with SI. Using 2016 data from the National Health and Aging Trends Study (NHATS) linked to Medicare claims, we calculated home time for sampled community-dwelling older adults using the HDAH measure. We operationally defined SI as having a diagnosis code in Medicare claims for at least one of nine severe chronic conditions identified by the Dartmouth Atlas. We conducted multivariate analyses to explore how a SI diagnosis (independent variable) is associated with home time (dependent variable). Covariates included gender, age, household composition, census division, and health and well-being (including self-reported health, number of chronic conditions, psychological symptoms, and subjective well-being). In unadjusted analyses, we found the overall community-dwelling sample population had a mean of 361.9 HDAH, while those with SI had a mean of 353.6 HDAH. A Poisson regression showed that community-dwelling older adults with SI had statistically significant fewer HDAH compared to those without SI (β=-0.03 fewer HDAH; CI:-0.03, -0.02). None of the covariate effects were statistically significant. This work furthers our understanding of opportunities to enhance service delivery systems for community-dwelling older adults with SI, a population not necessarily at the end-of-life but one that is vulnerable to frequent, intensive, high-cost health system encounters.

